# Protective Effects of Cannabidiol on Chemotherapy-Induced Oral Mucositis via the Nrf2/Keap1/ARE Signaling Pathways

**DOI:** 10.1155/2022/4619760

**Published:** 2022-05-25

**Authors:** Lin Li, Yaowei Xuan, Biao Zhu, Xing Wang, Xiaoyu Tian, Lisheng Zhao, Yan Wang, Xiaoxia Jiang, Ning Wen

**Affiliations:** ^1^Medical School of Chinese PLA, Beijing 100853, China; ^2^Department of Stomatology, The First Medical Centre, Chinese PLA General Hospital, Beijing 100853, China; ^3^Beijing Institute of Basic Medical Sciences, Beijing 100850, China

## Abstract

Oral mucositis (OM) is a common complication during chemotherapy characterized by ulceration, mucosa atrophy, and necrosis, which seriously interferes with nutritional intake and oncotherapy procedures among patients. However, the efficacy of current treatments for OM remains limited. Cannabidiol (CBD) is a natural cannabinoid with multiple biological activities, including antioxidant and anti-inflammatory potential. In this study, we aimed to investigate the chemopreventive effects and mechanisms of CBD in protecting C57BL/6N mice and human oral keratinocytes (HOK) from 5-fluorouracil- (5-FU-) induced OM. Here, we found that CBD alleviated the severity of 5-FU-induced OM in mice, including improved survival, decreased body weight loss, reduced ulcer sizes, and improved clinical scores. Histologically, CBD restored epithelial thickness and normal structure in tongue tissues. Meanwhile, CBD attenuated reactive oxygen species (ROS) overproduction and improved the antioxidant response, suppressed the inflammatory response, promoted the proliferation of epithelial cells, and inhibited 5-FU-induced apoptosis. *In vitro*, consistent outcomes showed that CBD suppressed cellular ROS levels, enhanced antioxidant ability, reduced inflammatory response, promoted proliferation, and inhibited apoptosis in 5-FU-treated HOK cells. In particular, CBD upregulated the expression levels of antioxidant enzymes, heme oxygenase-1 (HO-1) and NAD(P)H quinine oxidoreductase 1 (NQO1), by increasing the expression and nuclear translocation of nuclear factor erythroid 2-related factor 2 (Nrf2) and decreasing Kelch-like ECH-associated protein 1 (Keap1). Notably, the Nrf2 inhibitor ML385 reversed the protective effect of CBD. Nrf2-siRNA transfection also significantly blunted the antioxidant effect of CBD in *in vitro* OM model. Collectively, our findings suggested that CBD protected against 5-FU-induced OM injury at least partially via the Nrf2/Keap1/ARE signaling pathways, highlighting the therapeutic prospects of CBD as a novel strategy for chemotherapy-induced OM.

## 1. Introduction

Oral mucositis (OM) is one of the most common and serious complications under standard dose chemotherapy, radiotherapy, and pretreatment of hematopoietic stem cell transplantation [1, 2] and refers to pathological changes in oral mucosa, including erythema, ulceration, and necrosis after oncotherapy [3]. For patients with head and neck tumors, the incidence of OM is almost 100% [1, 4, 5]. Pain, infection, and difficulty eating affect life quality and oncotherapy prognosis [6]. However, current treatments focused on symptom relief have insufficient effectiveness [1]. The FDA-approved drug for OM, recombinant human keratinocyte growth factor *palifermin*, is also limited by its high cost and circumscribed efficacy [7].

Oral mucosa, as human organism gateway, is a highly responsive environment susceptible to triggering factors [8]. Chemotherapeutic drugs, as trigger factors, inducing oxidative stress and inflammatory response are increasingly recognized as hallmarks in the development and pathologies of OM [2, 9]. Excess reactive oxygen species (ROS) production and DNA damage caused by chemotherapy are the initial factors [10], resulting in epithelial injury and apoptosis, amplifying signals that activate inflammation and oxidative stress-related pathways [11]. Submucosal signals inhibit epithelial proliferation and migration and finally lead to unhealed mucosa [10]. Therefore, it is reasonable to hypothesize that attenuating overburdened oxidative stress and excess inflammatory response may be promising strategies for managing this disease.

Plant-derived bioactives, often called phytochemicals, have high efficacy in the treatment of oral mucosal lesions for a long history [8]. Cannabinoids, in particular, show great promise in protecting epithelial barriers due to their beneficial properties [12]. Cannabidiol (CBD) is currently the best studied nonpsychotropic cannabinoid with multiple targets. Based on extensive pharmacological effects, CBD functions as a potent antioxidant and anti-inflammatory agent in various diseases, including Parkinson's disease, Alzheimer's disease, epilepsy, dermatitis, and ischemia-reperfusion injury [13, 14]. In skin keratinocytes, CBD can reduce the expression levels of proinflammatory cytokines and protect against ultraviolet-related oxidative stress injury [15]. Recently, CBD was reported to increase antioxidant enzyme expression to promote endothelial cell survival [16]. CBD was also confirmed to have clinical evidence of antiepileptic effects by human clinical trials [14]. Alternatively, Cuba et al. found that CBD seemed to favor OM mice, but limited data was observed [17]. Despite these findings, the effects and mechanisms of CBD on chemotherapy-induced OM in mice and human oral keratinocytes (HOK) have not been determined in detail previously.

The present study is aimed at investigating the efficacy and possible mechanisms of CBD for OM protection *in vivo* and *in vitro*. In our study, C57BL/6N mice and HOK cells were treated with high doses of 5-fluorouracil (5-FU) to induce chemotherapy-related toxicity, and we found that CBD alleviated 5-FU-induced OM, improved cellular antioxidant ability, and suppressed the inflammatory response in mice and HOK. The Nrf2/Keap1/ARE signaling pathways may be the possible mechanism for the beneficial effects of CBD. In general, CBD could be considered as a promising strategy for chemotherapy-related OM.

## 2. Materials and Methods

### 2.1. Chemotherapy-Induced Oral Mucositis Models

All animal experiments were approved by the Institutional Animal Care and Use Committee of Chinese People's Liberation Army General Hospital. Six-week-old C57BL/6N mice were purchased from Beijing Vital River Laboratory Animal Technology. All mice were housed in a standard animal facility under controlled temperature (21°C) and photoperiod (12 hrs light/12 hrs dark) with free access to water and food.

After adaptive feeding for 2 weeks, the mice were randomly divided into the following 5 groups: (i) vehicle; (ii) 5-FU alone (50 mg/kg); and (iii) 5-FU (50 mg/kg) plus CBD treatment (3, 10, and 30 mg/kg) (*n* = 6). Except for the vehicle group, the mice received 5-FU (Sigma–Aldrich, USA) injection, once daily for 5 continuous days. CBD (CATO, USA) were intraperitoneally administered half an hour before 5-FU injection. The vehicle and 5-FU alone groups were treated with the solvent of CBD, 2% Tween 80+3% DMSO+95% saline, as placebo. Survival conditions and body weight were recorded daily. On Days 4, 7, and 10, the mice were euthanized with 2,2,2-tribromoethanol (Sigma–Aldrich, USA), and samples were collected for subsequent detection.

### 2.2. Clinical Score

The fecal and coat scores were assessed on Day 4 as markers of the systemic conditions of chemotherapy-treated mice, which were conducted by two different researchers in a blind manner. Descriptions of fecal score and coat score are shown in Tables S2 and S3 [3, 18].

### 2.3. Histological Evaluation

On Day 10, the harvested tongue tissues were stained with 1% toluidine blue dye for 1 min and then washed with 1% acetic acid and PBS to reveal the ulcer sizes on the mouse tongues (*n* = 3). The tongues were then collected and fixed with 4% paraformaldehyde for 5 days. After dehydration and paraffin embedding, the tissues were sliced into 5 *μ*m thick sections for subsequent histological staining. H&E staining was performed. For immunohistochemical (IHC) staining, primary antibodies including Ki67 (#12202, dilution: 1 : 200, Cell Signaling Technology, USA), nuclear factor erythroid 2-related factor 2 (Nrf2, A0674, dilution: 1 : 200, ABclonal, China), and Kelch-like ECH-associated protein 1 (Keap1, A1820, dilution: 1 : 200, ABclonal, China) were incubated overnight, and secondary antibodies were then incubated for 30 min on the second day, followed by DAB staining. After using hematoxylin, the nuclei were restained. Terminal deoxynucleotidyl transferase-mediated dUTP nick-end labeling (TUNEL) assay was performed using a TUNEL Staining Kit (C1098, Beyotime, China) following the manufacturer's protocol. The sections were observed under a microscope (Olympus, Japan) and photographed. ImageJ software (National Institutes of Health, Bethesda, MD, USA) was used to quantify ulcer sizes, positive cells, and epithelial thickness.

### 2.4. Enzyme-Linked Immunosorbent Assay (ELISA)

After collecting mouse blood on Day 4, the samples were placed at room temperature for 2 hrs and then centrifuged at 4000 r/min for 10 min at 4°C to separate the serum. According to the instructions of the ELISA kit (Dakewe Bioengineering, China), we calculated the concentrations of inflammatory cytokines, tumor necrosis factor-*α* (TNF-*α*) and interleukin 6 (IL-6).

### 2.5. ROS Level Assay

On Day 7, the harvested tongue samples (*n* = 3) were immediately embedded in OCT (TISSUE-TEK; Sakura Finetek USA, Inc.) and cut into 10 *μ*m thick sections by a frozen slicer. The sections were stained with 2′,7′-dichlorodihydrofluorescein diacetate (DCFH-DA) solution (S0033, Beyotime, China) at a final concentration of 10 *μ*M. After incubation at 37°C for 20 min, the sections were washed with PBS for 3 times, observed and photographed under fluorescence microscopy (Olympus, Japan). For *in vitro* studies, DCFH-DA, as a fluorescent probe, was also used to assess intracellular ROS levels after 5-FU (10 *μ*g/mL), LPS (10 *μ*g/mL, Sigma–Aldrich), hydrogen peroxide (H_2_O_2_, 200 *μ*mol/L), and different concentrations of CBD (0.5, 2.5, and 5 *μ*M) intervention. HOK (#2610, ScienCell, USA) cells were seeded in 24-well plates (5 × 10^4^ per well). After 24 hrs of culture, the cells were pretreated with CBD for 12 hrs, and then, 5-FU (12 hrs), LPS (6 hrs), and H_2_O_2_ (10 min) were added. The following steps were the same as above. The mean fluorescence intensity was analyzed by ImageJ software.

### 2.6. In Vitro Models of Oral Mucositis

HOK cells were cultured in 1640 medium (Gibco, USA) containing 10% fetal bovine serum (FBS, Gibco, USA) and 1% penicillin-streptomycin in a humidified incubator at 37°C with 5% CO_2_. Corresponding to the *in vivo* experiments, 5-FU was applied to HOK cells to establish an *in vitro* model of chemotherapy-induced OM. In addition, LPS and H_2_O_2_ were also used, as classical reagents to induce inflammatory and oxidative stress environments in HOK cells, respectively.

### 2.7. Cell Proliferation Assay

A CCK-8 assay was used to detect the effects of reagents on cell viability and proliferation. HOK cells were inoculated in 96-well plates (5 × 10^3^ per well). First, cytotoxicity analysis was performed to determine the effects of different concentrations of CBD (0.1, 0.5, 2.5, 5, and 10 *μ*M), 5-FU (0.1, 1, 10, 20, 30, and 40 *μ*g/mL), LPS (0.5, 1, 5, 10, and 20 *μ*g/mL), and H_2_O_2_ (50, 100, 200, 300, and 400 *μ*mol/L) on HOK viability for 24 hrs and 48 hrs. Second, the cells were pretreated with CBD (0.5, 2.5, and 5 *μ*M) for 12 hrs and then treated with 10 *μ*g/mL 5-FU. After culture for 1, 3, and 5 days, 10 *μ*L CCK-8 solution (Lablead, China) and 90 *μ*L fresh medium were added to each well and incubated for 2 hrs at 37°C. Then, the absorbance at 450 nm was detected using a microplate reader (Rayto RT-6000, USA). The cell viability (%) was calculated as follows: [(A450 of the treated cells − A450 of the blank)/(A450 of vehicle group cells − A450 of the blank)] × 100%.

### 2.8. Cell Apoptosis Detection

Annexin V-FITC/PI staining was performed to detect the apoptosis rates of the treated HOKs using a Cell Apoptosis Detection Kit (UEland, China) as instructed by the manufacturer. Briefly, HOKs were pretreated with different concentrations of CBD (0.5, 2.5, and 5 *μ*M) for 12 hrs and then incubated with 10 *μ*g/mL 5-FU for 12 hrs. After collecting 5 × 10^5^ cells, the apoptosis rates (%) among different groups were examined by flow cytometry (BD Celesta, USA) and finally analyzed using FlowJo software.

### 2.9. Wound Healing Assay

HOK were seeded in 6-well plates (6 × 10^5^ per well). When HOK cells reached approximately 90-100% confluence, we scratched the bottom of the culture plates with a 200 *μ*L pipette tip (Axygen, USA) to achieve a straight wound. The cells were washed with PBS and incubated with different serum-free cell medium for 12 and 24 hrs. Photos were taken, and cell migration was recorded under a microscope. The healing areas were analyzed by ImageJ software. The healing rates (%) at 12 hrs and 24 hrs were calculated as follows: [(scratch area at 0 h − scratch area at 12 or 24 hrs)/scratch area at 0 h] × 100%.

### 2.10. Immunofluorescence (IF) Staining

The expression levels and nuclear translocation of Nrf2 among groups were observed by laser scanning confocal microscopy (Olympus, Japan). We applied 4% paraformaldehyde to fix the cells, and then, 0.25% Triton X-100 was applied to permeabilize HOK cells, followed by blocking with 1% bovine serum albumin. Anti-Nrf2 antibody (red, ab31163, dilution: 1 : 100, Abcam, USA) and DAPI (C1002, Beyotime, China) for nuclear staining (blue) were used.

### 2.11. Small Interfering RNA (siRNA) Transfection

Nrf2-siRNA (GenePharma, China) was used to knock down the expression levels of Nrf2 in HOK, and Scramble-siRNA were also added as control. Nrf2-siRNA and Scramble-siRNA were transfected into HOKs with Lipofectamine 2000 transfection reagent (Thermo Fisher, USA) as instructed. After transfection, the mRNA expression levels of Nrf2 were detected to verify the knockdown efficiency by qRT-PCR.

### 2.12. Quantitative Real-Time Polymerase Chain Reaction (qRT-PCR)

qRT-PCR was used to detect the mRNA expression levels of inflammation-, oxidative stress-, and apoptosis-related genes in mouse tongue tissue or HOK cells samples. *β*-Actin was used as the internal control. Total RNA extraction and cDNA synthesis were conducted as described in a previous study [19]. qRT-PCR (Sangon Biotech, China) was performed with the following procedures: 95°C for 3 min; 95°C for 15 sec, 60°C for 15 sec, 72°C for 15 sec; and 40 cycles. The 2^-*ΔΔ*Ct^ method was adopted to analyze the relative mRNA expression. Primer sequences were designed by Sangon Biotech, China, and shown in Table S1.

### 2.13. Western Blotting

Western blotting was performed to detect the protein expression levels. The detailed procedures were described in a previous study [20]. The primary antibodies used were HO-1 (10701-1-AP, dilution: 1 : 2000, Proteintech, USA), NQO1 (ab34173, dilution: 1 : 1000, Abcam, USA), Nrf2 (ab137550, dilution: 1 : 1000, Abcam, USA), Keap1 (GTx60660, dilution: 1 : 1000, GeneTex, USA), Lamin B (BA1228, dilution: 1 : 1000, Boster, China), and *β*-actin (BM0627, dilution: 1 : 200, Boster, China). Blots were quantified using ImageJ software.

### 2.14. Statistical Analysis

Experimental data are expressed as the means ± standard deviation (mean ± SD). Statistical analysis was performed using SPSS (version 23.0, USA) and GraphPad Prism Software (Version 7.0, Inc., La Jolla, CA, USA). One-way analysis of variance (ANOVA) and Tukey's multiple-comparisons test were performed for data analysis. *P* < 0.05 was considered statistically significant.

## 3. Results

### 3.1. CBD Alleviates the Severity of Chemotherapy-Induced OM in Mice

**Figure 1:**
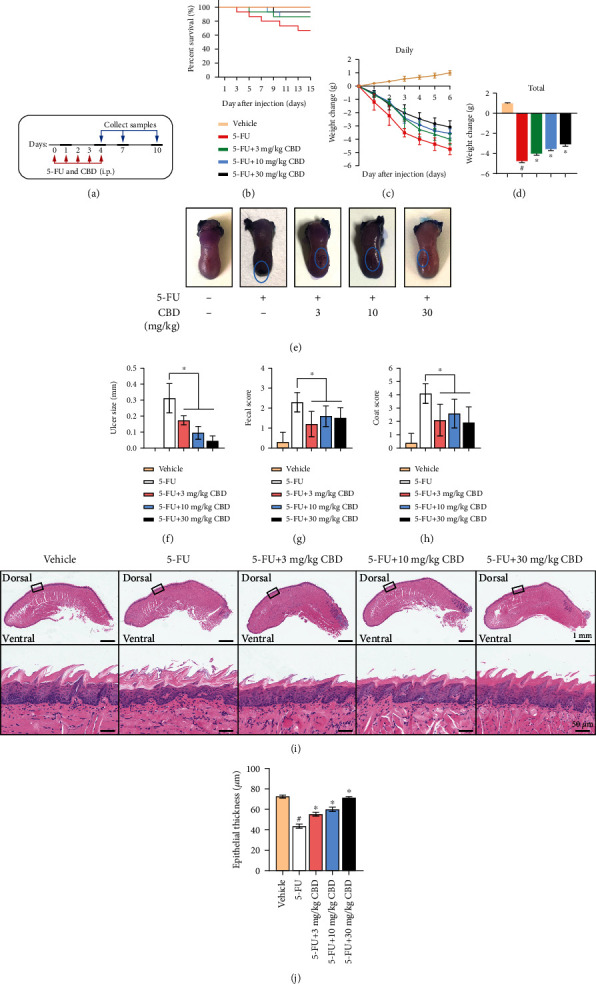
CBD alleviates the severity of chemotherapy-induced OM in mice. (a) Flow diagram of the 5-FU-induced OM model and CBD treatment in mice. (b) Survival curves of mice exposed to drug intervention. (c) Daily body weight loss. (d) Total weight loss on Day 6. (e) Representative images of the ulcer in tongues. (f) Measurements of ulcer sizes. (g) Fecal scores of the mice on Day 4. (h) Coat scores of the mice on Day 4. (i) Representative morphological exhibition of the tongue tissues and epithelium among groups as measured by HE staining. Upper scale bar, 1 mm. Lower scale bar, 50 *μ*m. (j) Epithelial thickness quantification of tongue tissues based on HE staining. Data are expressed as the means ± SD. ^#^*P* < 0.05*vs.* vehicle; ^∗^*P* < 0.05*vs.* 5-FU alone group.

OM was induced in C57BL/6N mice by 5-FU intervention for 5 consecutive days, and CBD (3, 10, and 30 mg/kg) was administered a half-hour prior (Figure 1(a)). First, 5-FU-induced toxicity significantly reduced mouse survival and continuously aggravated weight loss, whereas CBD administration improved survival (Figure 1(b)) and reduced weight reduction in mice exposed to 5-FU toxicity. CBD significantly increased the daily and total body weight of 5-FU-exposed mice in a dose-dependent manner (Figures 1(c) and 1(d)).

Clinically, untreated mice developed severe ulcers due to the toxicity of 5-FU. Meanwhile, worse fecal and coat score results showed similar outcomes: a high dose of 5-FU increased systemic adverse reactions. Of note, we observed that CBD treatment decreased the severity and sizes of the ulcers (Figures 1(e) and 1(f)) and improved the clinical scores and systemic conditions of the mice (Figures 1(g) and 1(h)).

Next, we evaluated the pathology in tongue tissues after 5-FU and CBD intervention. Morphological evaluation of the tongue tissues and epithelium was performed using HE staining (Figure 1(i)). Histopathological images showed that the epithelium was atrophic and disordered in untreated 5-FU group mucosa, including disorganized epithelial structure and reduced epithelial thickness. However, CBD increased epithelial thickness and restored the normal epithelial structure in a dose-dependent manner. Quantification of the epithelial thickness based on histologic examination is shown in Figure 1(j).

### 3.2. CBD Defends against Oxidative Stress and Inflammatory Response in a Mouse OM Model

**Figure 2:**
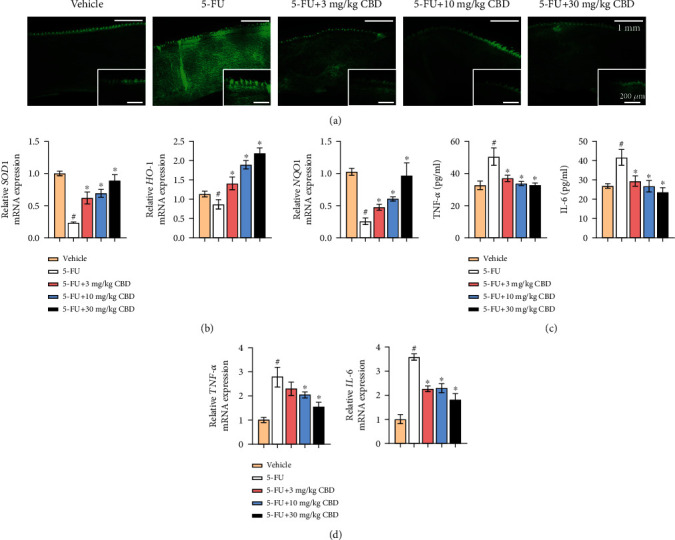
CBD defends against oxidative stress and inflammatory response in a mouse OM model. (a) Representative images of DCFH-DA staining for ROS detection. Upper scale bar, 1 mm. Lower scale bar, 200 *μ*m. (b) mRNA expression levels of *SOD1*, *HO-1*, and *NQO1* in mouse tongue tissues as measured by qRT-PCR. (c) Serum TNF-*α* and IL-6 levels were detected by ELISA. (d) mRNA expression levels of *TNF-α* and *IL-6* in mouse tongue tissues were detected by qRT-PCR. *β*-Actin was used as the internal control. Data are expressed as the means ± SD. ^#^*P* < 0.05*vs.* vehicle; ^∗^*P* < 0.05*vs.* 5-FU alone group.

Enhanced oxidative stress is closely associated with OM pathology, and ROS accumulation is the initial factor [10]. First, DCFH-DA detection was used to evaluate ROS levels in tongue tissues. 5-FU intervention indeed increased the ROS level in the mouse tongue, while the ROS levels in CBD-treated mice were significantly decreased (Figure 2(a)). Additionally, using qRT-PCR, we evaluated the mRNA expression levels of superoxide dismutase 1 (*SOD1*), heme oxygenase-1 (*HO-1*), and NAD(P)H quinine oxidoreductase 1 (*NQO1*), important antioxidants, and cellular protective enzymes in the oxidative stress process. CBD intervention contributed to improving the mRNA expression levels of *SOD1*, *HO-1*, and *NQO1* in a dose-dependent manner, which were suppressed by chemotherapy (Figure 2(b)). These results confirmed that CBD protected OM mice against oxidative stress.

Chemotherapy results in an increase in proinflammatory cytokines, including TNF-*α* and IL-6, which not only damage mucosal tissue but also amplify primary destruction by a feedback loop [10]. ELISA was used to detect inflammatory cytokine levels in mouse serum on Day 4. CBD reduced TNF-*α* and IL-6 levels, which were increased in 5-FU-treated mice (Figure 2(c)). In parallel, CBD significantly decreased the mRNA expression levels of *TNF-α* and *IL-6* in tongue tissue by qRT-PCR (Figure 2(d)), which was consistent with the ELISA results.

### 3.3. CBD Promotes Epithelial Cell Proliferation and Inhibits 5-FU-Induced Apoptosis in Mouse Tongue Tissue

**Figure 3:**
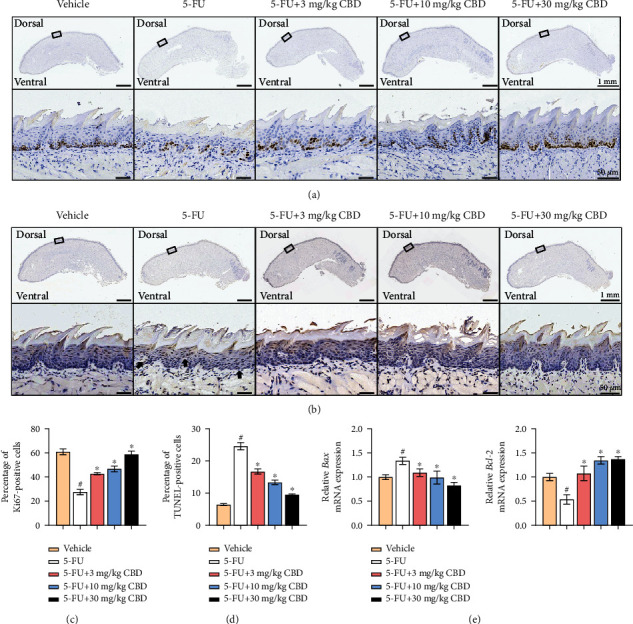
CBD promotes epithelial cell proliferation and inhibits 5-FU-induced apoptosis in mouse tongue tissue. (a) Representative IHC staining of Ki67 antibody for proliferation of mucosal epithelial cells. Upper scale bar, 1 mm. Lower scale bar, 50 *μ*m. (b) Representative images of TUNEL staining for apoptosis of mucosal epithelial cells. Upper scale bar, 1 mm. Lower scale bar, 50 *μ*m. (c) Semiquantification of Ki67-positive epithelial cells in (a). (d) Semiquantification of TUNEL-positive epithelial cells in (b). (e) mRNA expression levels of *Bax* and *Bcl-2* in mouse tongue tissues as measured by qRT-PCR. *β*-Actin was used as the internal control. Data are expressed as the means ± SD. ^#^*P* < 0.05*vs.* vehicle; ^∗^*P* < 0.05*vs.* 5-FU alone group.

Epithelial cell apoptosis is one of the major pathologies in chemotherapy-induced OM and is caused by excess ROS generation [10]. IHC-stained dorsal and ventral tongue sections using Ki67 and TUNEL staining were used to investigate the proliferation and apoptosis of mucosal basal layer cells, respectively. The counts of Ki67-positive epithelial cells were significantly reduced, while the TUNEL-positive cells were increased in the tongue tissues subjected to 5-FU intervention, indicating that 5-FU-generated toxicity inhibited basal layer cell proliferative capacity. In contrast, CBD treatment enhanced Ki67-positive epithelial cellularity and reduced TUNEL-labeled apoptotic cells (Figures 3(a) and 3(b)), confirming the protective effects of CBD against 5-FU-induced cytotoxicity. Quantitative analysis of positive cellularity is shown in Figures 3(c) and 3(d).

The mRNA expression level detection of apoptosis-related genes in tongue tissue showed that CBD decreased the expression level of *Bax* and increased the expression of *Bcl-2* compared with mice receiving only 5-FU treatment (Figure 3(e)). Collectively, these findings demonstrated that CBD effectively promoted the proliferation of epithelial cells and inhibited 5-FU-induced apoptosis.

### 3.4. CBD Enhances HOK Proliferative Capacity and Cell Migration in In Vitro OM Models

**Figure 4:**
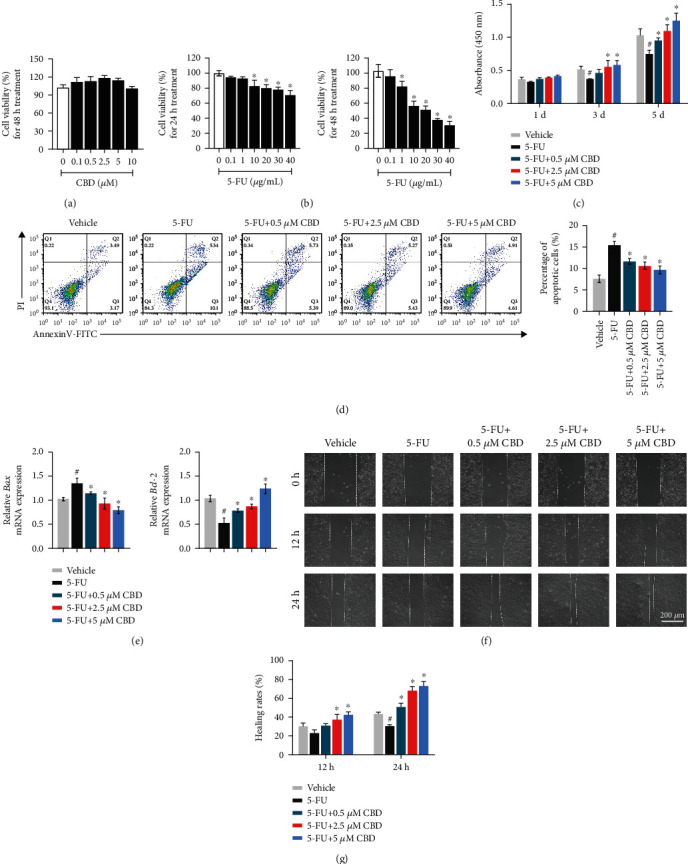
CBD enhances HOK proliferative capacity and cell migration in *in vitro* OM models. (a) Cell viability of HOK cells exposed to different concentrations of CBD (0.1-10 *μ*M) for 48 hrs was detected by CCK-8 assay. (b) HOK viability after 24 hrs and 48 hrs exposure to 5-FU (0.1-40 *μ*g/mL). ^∗^*P* < 0.05*vs.* vehicle group. (c) CCK-8 results showing the proliferation of HOK cells after exposure to 5-FU (10 *μ*g/mL) and CBD (0.5, 2.5, and 5 *μ*M) for 1, 3, and 5 days. (d) Flow cytometry results of HOK cells treated with 5-FU and CBD to detect the apoptosis rates. (e) mRNA expression levels of *Bax* and *Bcl-2* in HOK cells as measured by qRT-PCR. *β*-Actin was used as the internal control. (f) Representative images of wound healing assays for HOK migration at 0 h, 12 hrs, and 24 hrs. Scale bar, 200 *μ*m. (g) Quantitative analysis of the healing rates. Data are expressed as the means ± SD. ^#^*P* < 0.05*vs.* vehicle; ^∗^*P* < 0.05*vs.* 5-FU alone group.

Subsequently, we wondered whether CBD could play a protective role in *in vitro* OM models. Therefore, 5-FU was applied to HOK to induce an oral mucositis environment. First, the viability of HOK cells exposed to different concentrations of CBD and 5-FU for 24 hrs and 48 hrs was detected using CCK-8 assay. The results showed no significant differences in absorbance values among different concentrations of CBD after 48 hrs (Figure 4(a)), indicating that CBD had no obvious toxic effects on HOK vitality at the tested concentrations. However, 5-FU reduced HOK cell viability in a concentration-dependent manner (*P* < 0.05) (Figure 4(b)). Therefore, the 10 *μ*g/mL 5-FU concentration was determined for inhibiting 50% viability (IC50) after 48 hrs of culture. Meanwhile, the concentrations of CBD used were based on the preexperiments that CBD obviously decreased the inflammatory cytokine expression at 0.5, 2.5, and 5 *μ*M final concentrations (Figure S1(a)).

After, the cytoprotective effects of CBD on the proliferation of 5-FU-exposed HOK cells were investigated by the CCK-8 assay. The cells were divided into five groups: vehicle, 5-FU, and 5-FU plus CBD (0.5, 2.5, and 5 *μ*M), and the absorbance on Days 1, 3, and 5 was measured. As shown in Figure 4(c), the proliferation of 5-FU-treated HOK cells was statistically inhibited on the 3rd day of culture. By comparison, absorbance values at 450 nm in the CBD intervention group were enhanced with statistical significance on the 3rd and 5th days.

Meanwhile, an Annexin V-FITC/PI assay was performed to analyze the apoptosis of HOK cells treated with 5-FU and CBD (Figure 4(d)). Flow cytometry results demonstrated that the proportion of apoptotic cells in the 5-FU group was increased, and different concentrations of CBD reversed this increase (*P* < 0.05). Consistent outcomes were observed by qRT-PCR, as CBD reversed the decreased mRNA expression level of *Bcl-2* and increased expression of *Bax* in the 5-FU group (Figure 4(e)). In conclusion, these findings indicated that CBD promoted proliferation and inhibited apoptosis in 5-FU-induced HOK cells.

Cell migration potentiality is a critical event in the healing of injured epithelial tissue. A scratch assay was conducted to investigate the migration ability of HOK cells after 5-FU and CBD intervention. We observed that the healing rates of HOKs were the lowest in serum-free medium containing 5-FU alone, indicating that cell migration was inhibited by 5-FU toxicity, whereas pretreatment with CBD recovered the healing rates of injured HOKs (Figure 4(f)). The healing rates at 12 hrs and 24 hrs are shown in Figure 4(g).

### 3.5. CBD Reduces Intracellular ROS Production and Inflammatory Cytokine Levels In Vitro

**Figure 5:**
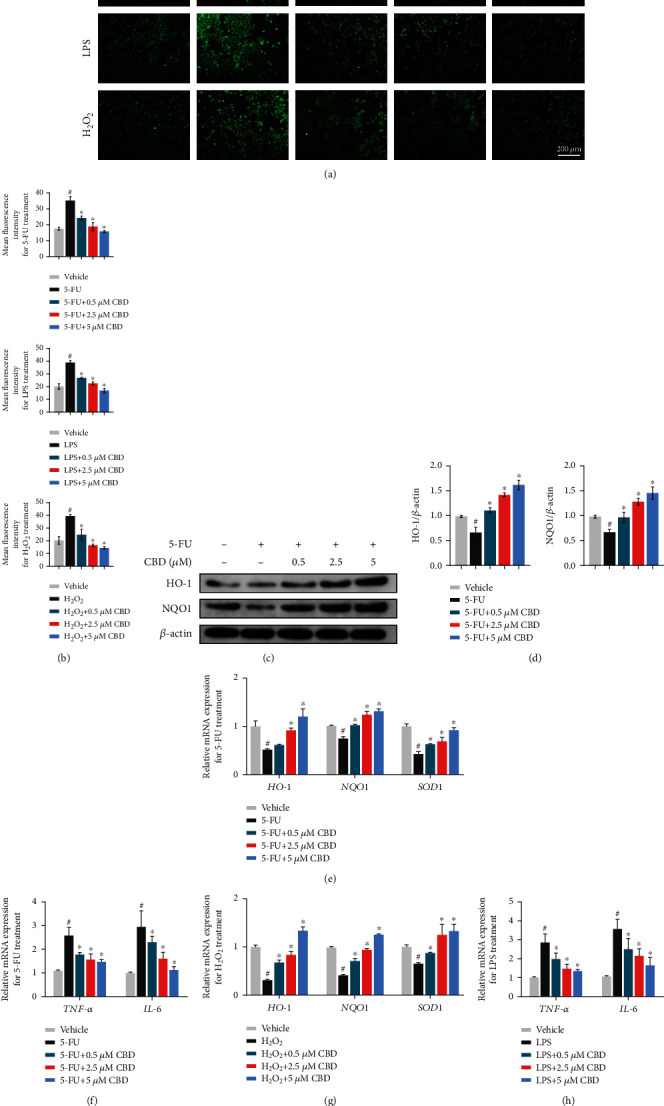
CBD reduces intracellular ROS production and inflammatory cytokine levels. (a) DCFH-DA staining of the treated HOK cells to evaluate intracellular ROS generation, which was determined following 5-FU (10 *μ*g/mL, 12 hrs), LPS (10 *μ*g/mL, 6 hrs), and H_2_O_2_ (200 *μ*mol/L, 10 min) intervention and compared with that of vehicle or CBD pretreatment (0.5, 2.5, and 5 *μ*M). Scale bar, 200 *μ*m. (b) Quantitative analysis of the mean fluorescence intensity in (a). (c) Western blotting was performed to detect the protein levels of HO-1 and NQO1 between groups. *β*-Actin was used as the internal control. (d) Grayscale analysis of the blots. (e) HOK cells were pretreated with CBD (0.5, 2.5, and 5 *μ*M) for 12 hrs, and then, 5-FU (10 *μ*g/mL) was added and incubated for another 12 hrs. qRT-PCR was performed to detect the mRNA expression levels of *HO-1*, *NQO1*, and *SOD1*. (f) The interventions were the same as (e), and the mRNA expression levels of *TNF-α* and *IL-6* were detected. (g) mRNA expression levels of *HO-1*, *NQO1*, and *SOD1* after H_2_O_2_ (200 *μ*mol/L, 12 hrs) and CBD treatment. (h) mRNA expression levels of *TNF-α* and *IL-6* after LPS (10 *μ*g/mL, 12 hrs) and CBD intervention. *β*-Actin was used as the internal control of qRT-PCR. Data are expressed as the means ± SD. ^#^*P* < 0.05*vs.* vehicle; ^∗^*P* < 0.05*vs.* the intervention alone group.

To confirm the antioxidant potential of CBD *in vitro*, 5-FU, LPS, and H_2_O_2_ were used to induce excess cellular ROS release in HOK cells. Then, we found that the mean fluorescence intensities of the CBD treatment groups were significantly reduced compared with those of the 5-FU, LPS, and H_2_O_2_ alone groups (Figures 5(a) and 5(b)). Furthermore, we detected the protein expression levels of HO-1 and NQO1 by western blotting. The results showed that 5-FU intervention reduced the protein levels of HO-1 and NQO1, while CBD reversed this decline in a dose-dependent manner (Figures 5(c) and 5(d)). The original blots are shown in Figure S2.

Next, the mRNA expression levels of *HO-1*, *NQO1*, and *SOD1* were detected by qRT-PCR after 5-FU (10 *μ*g/mL, 12 hrs) and H_2_O_2_ (200 *μ*mol/L, 12 hrs) intervention. CBD pretreatment significantly upregulated the mRNA expression levels of these genes, which was consistent with the western blotting results (Figures 5(e) and 5(g)). H_2_O_2_ concentration was determined for inhibiting 50% viability (IC50) of HOK at 48 hrs of culture (Figure S1(b)). The mRNA expression levels of *SOD1* and *HO-1* in HOK under different concentrations of H_2_O_2_ are shown in Figure S1(c). These findings collectively indicated the beneficial effects of CBD against excess oxidative stress *in vitro*.

Consistent with the *in vivo* experiments, we used 5-FU (10 *μ*g/mL, 12 hrs) and LPS (10 *μ*g/mL, 12 hrs) to treat HOK to simulate the inflammatory environments of chemotherapy-induced OM *in vitro*. As expected, pretreatment with CBD reversed the increase of inflammatory cytokine expression levels by 5-FU and LPS intervention (Figures 5(f) and 5(h)). LPS concentration was determined for inhibiting 50% viability (IC50) at 48 hrs (Figure S1(d)). The mRNA expression levels of *TNF-α* and *IL-6* after 5-FU (1-20 *μ*g/mL) or LPS (0.5-20 *μ*g/mL) intervention are shown in Figures S1(e) and S1(f).

### 3.6. CBD Activates Nrf2/Keap1/ARE Signaling Pathways in OM

**Figure 6:**
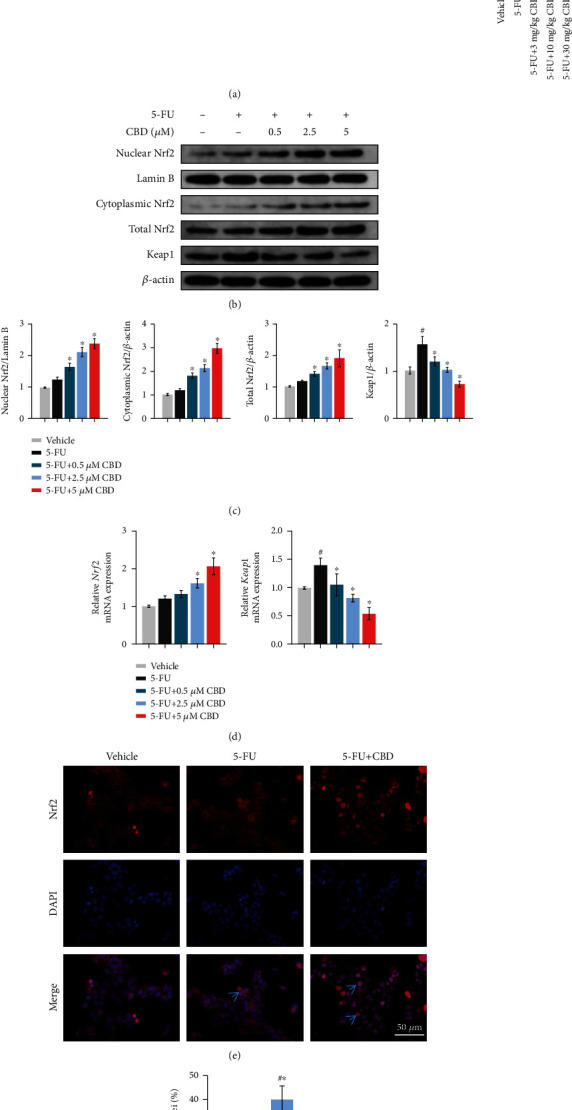
CBD activates Nrf2/Keap1/ARE signaling pathways. (a) Representative IHC staining images of Nrf2 and Keap1 expression in mouse tongue tissues and quantification of positively stained epithelial cells. Positively stained cells are indicated by arrows. Scale bar, 50 *μ*m. (b) Western blotting analysis of nuclear Nrf2, cytoplasmic Nrf2, total Nrf2, and Keap1 protein levels in HOK cells. *β*-Actin was used as the internal control. (c) Grayscale analysis of the blots. (d) mRNA expression levels of *Nrf2* and *Keap1* after CBD treatment. (e) Detection of Nrf2 (red) expression and DAPI (blue) in HOKs treated with 5-FU and CBD (5 *μ*M) by IF microscopy. Positive Nrf2 staining for nuclear translocation is indicated by arrows. Scale bar, 50 *μ*m. (f) Semiquantification of the Nrf2-positive nuclei. Data are expressed as the means ± SD. ^#^*P* < 0.05*vs.* vehicle; ^∗^*P* < 0.05*vs.* 5-FU alone group.

The Nrf2/Keap1/antioxidant responsive element (ARE) signaling pathways are crucial mechanisms against the oxidative stress process for OM defense. Previous studies revealed that Nrf2 is a downstream target of CBD [21, 22]. To examine whether CBD activated the Nrf2/ARE signaling pathways during OM pathology, we detected the expression of Nrf2 and Keap1 in tongue tissues and HOK cells. IHC staining revealed that Nrf2-positive epithelial cells in the CBD groups were significantly increased. In contrast, Keap1 accumulation was gradually attenuated in tongue mucosa with increasing CBD doses (Figure 6(a)). Interestingly, Nrf2 expression in tongue tissue exposed to 5-FU alone was also increased slightly, indicating that 5-FU could also activate the Nrf2. The expression of Keap1 was also upregulated by 5-FU. To some extent, CBD promoted Keap1 degradation and improved Nrf2 protein stability. Moreover, HO-1 and NQO1, key target genes transcriptionally regulated by Nrf2, were upregulated by CBD in OM models (Figure 5(c)).

Nrf2 is usually located in the cytoplasm and is activated after entering the nucleus. To investigate how CBD affects Nrf2 activation during oxidative stress, we first extracted the proteins in HOK nuclei, cytoplasm, and total protein, respectively. Consistent with the *in vivo* data, western blotting analysis showed that CBD treatment significantly promoted the protein expression of Nrf2 in both the nucleus and cytoplasm (Figures 6(b) and 6(c)); the original blots are shown in Figure S3. Second, qRT-PCR exhibited that the mRNA expression levels of Nrf2 and Keap1 were consistent with western blot (Figure 6(d)). Moreover, the IF staining results showed that the Nrf2 protein was highly localized in the nucleus after CBD intervention (Figures 6(e) and 6(f)). These findings revealed the role of CBD in Nrf2 activation by promoting the upregulation and nuclear translocation.

### 3.7. CBD Plays a Protective Effect on the OM via the Nrf2/Keap1/ARE Pathway

**Figure 7:**
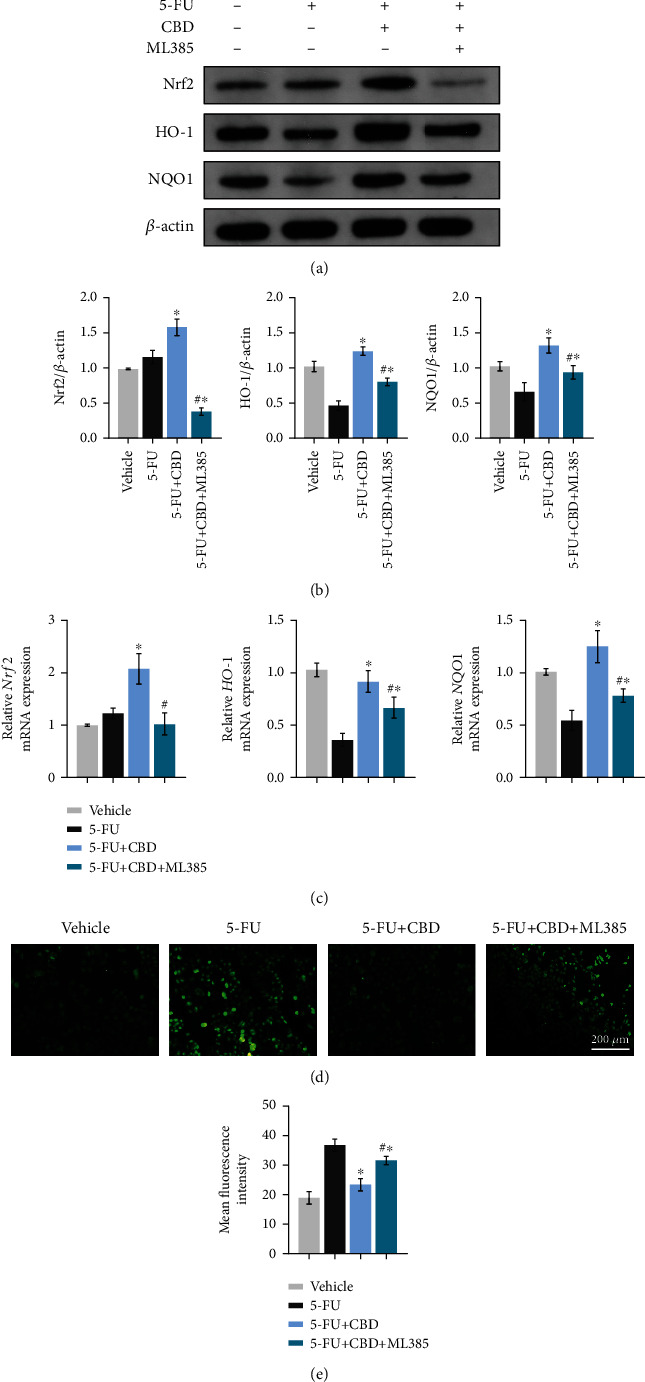
ML385 blunted the antioxidant effects of CBD in *in vitro* OM model. (a) HOK cells were pretreated with CBD for 12 hrs, and then, 5-FU and ML385 (10 *μ*M) were added and incubated for another 12 hrs. Western blotting of Nrf2, HO-1, and NQO1 protein levels was performed. *β*-Actin was used as the internal control. (b) Grayscale analysis of the blots. (c) HOK was harvested to measure the mRNA expression levels of *Nrf2*, *HO-1*, and *NQO1* using qRT-PCR. *β*-Actin was used as the internal control. (d) DCFH-DA staining of the treated HOK cells to evaluate intracellular ROS generation. Scale bar, 200 *μ*m. (e) Quantitative analysis of the mean fluorescence intensity in (d). Data are expressed as the means ± SD. ^∗^*P* < 0.05*vs.* the 5-FU alone group; ^#^*P* < 0.05*vs.* the 5-FU+CBD group.

To further explore whether the Nrf2/Keap1/ARE signaling pathways are the underlying mechanism for CBD-mediated protective effects in OM, HOK cells were treated with ML385 (an inhibitor of Nrf2). First, western blotting illustrated that ML385 successfully counteracted the expression level of Nrf2, which was increased by CBD. Second, ML385 significantly blunted the protein expression of HO-1 and NQO1 in CBD-treated HOK cells (Figures 7(a) and 7(b)). The original blots are shown in Figure S4. Consistent with the western blotting results, qRT-PCR verified the decreased mRNA expression levels of *Nrf2*, *HO-1*, and *NQO1* after ML385 application (Figure 7(c)). We also observed that ML385 reversed the decline of ROS overproduction (Figures 7(d) and 7(e)). However, the enhanced effects of the target genes were not completely inhibited by the addition of the Nrf2 inhibitor ML385.

**Figure 8:**
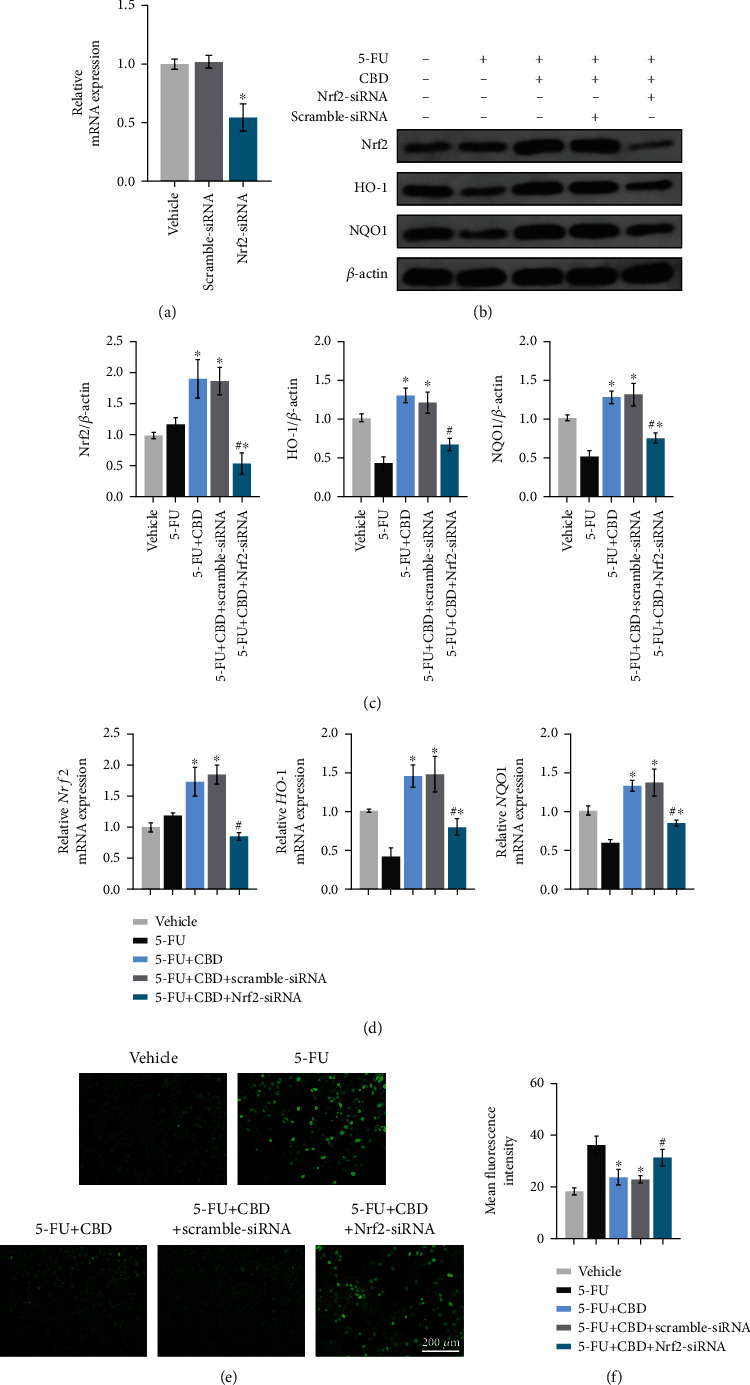
CBD plays protective effects on OM via the Nrf2/Keap1/ARE pathway. HOK cells were transfected with Nrf2-siRNA and Scramble-siRNA, respectively. After 6 hrs transfection, HOK cells were treated with 5-FU and CBD. Then, the cells were collected for the following analysis. (a) Knockdown efficiency of Nrf2 as measured by qRT-PCR. ^∗^*P* < 0.05*vs.* vehicle group. (b) Detection of Nrf2, HO-1, and NQO1 protein levels by western blotting. *β*-Actin was used as the internal control. (c) Grayscale analysis of the blots. (d) The mRNA expression levels of *Nrf2*, *HO-1*, and *NQO1* as measured by qRT-PCR. *β*-Actin was used as the internal control. (e) DCFH-DA staining. Scale bar, 200 *μ*m. (f) Quantitative analysis of the mean fluorescence intensity. Data are expressed as the means ± SD. ^∗^*P* < 0.05*vs.* the 5-FU alone group; ^#^*P* < 0.05*vs.* the 5-FU+CBD group.

Furthermore, HOK cells were transfected with Nrf2-siRNA and Scramble-siRNA, respectively. Transduction efficiency was confirmed by qRT-PCR, and Nrf2 expression was significantly knocked down after transfecting with Nrf2-siRNA (Figure 8(a)). As shown in western blot (Figures 8(b) and 8(c)), the increased expression of Nrf2 by CBD application was reversed when HOK were cocultured with Nrf2-siRNA, so was the expression of HO-1 and NQO1. The original blots are shown in Figure S5. In addition, the mRNA expression of *Nrf2*, *HO-1*, and *NQO1* was significantly blunted by Nrf2-siRNA, as compared with the Scramble-siRNA group and the 5-FU+CBD group (Figure 8(d)). DCFH-DA staining also indicated that Nrf2-siRNA transfection blocked the CBD-mediated ROS declination (Figures 8(e) and 8(f)). Together, CBD exerted protective effects on OM via the Nrf2/Keap1/ARE pathway, at least partially.

## 4. Discussion

Rapidly proliferating epithelial basal cells are often the target of antineoplastics due to the nonspecific action of chemotherapeutic agents and therefore lead to common toxicity events, such as mucositis, causing mucosal injury in the oral cavity and gastrointestinal tract [23]. With the increasing incidence of tumors, developing effective treatment options for OM to relieve unnecessary suffering of cancer patients is urgently needed.

**Figure 9:**
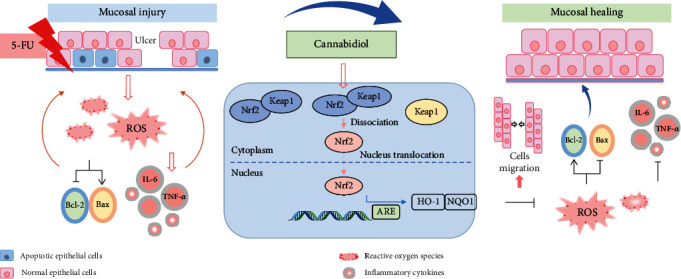
Protective effects of CBD against chemotherapy-induced OM. 5-FU, used in mice and HOK, triggered injury to basal layer epithelial cells and then induced OM. The pathologies of OM initiate from increased ROS levels, followed by an overburdened inflammatory response and cell apoptosis, resulting in the development of OM. CBD intervention reduces intracellular ROS overproduction, decreases excess inflammatory cytokine secretion, inhibits cell apoptosis, and promotes cell migration. Moreover, CBD treatment increases Nrf2 expression and promotes the translocation of Nrf2 from the cytoplasm to the nucleus, subsequently activating the Nrf2/Keap1/ARE pathways and upregulating the expression levels of downstream antioxidant enzymes to defend epithelial cells from 5-FU damage. Eventually, CBD administration promotes cell regeneration and alleviates OM.

5-FU is widely used for chemotherapy in clinical practice as a pyrimidine analogue and has been applied in animal experiments to simulate the chemotherapy environment [24]. In this study, we treated C57BL/6N mice and HOK cells with a high dose of 5-FU to induce chemotherapy-related mucositis. Our results demonstrated for the first time that CBD could effectively protect oral mucosa against 5-FU-induced toxicity *in vivo* and *in vitro*. The major findings are as follows. (1) CBD alleviated the severity of OM in 5-FU-induced mice and HOK. (2) CBD suppressed ROS overproduction, improved the antioxidant response, suppressed the excess inflammatory response, promoted epithelial cell proliferation, and inhibited cell apoptosis. (3) CBD increased Nrf2 levels and nuclear translocation. The Nrf2/Keap1/ARE signaling pathway might be a potential mechanism for CBD-treated OM recovery (Figure 9). Overall, CBD can be considered a promising strategy for the treatment of chemotherapy-associated OM.

Cannabinoids are naturally bioactive substances with ancient applications and controversial addiction properties [25]. However, unlike the main psychoactive substance, *Δ*-9-tetrahydrocannabinol (THC), CBD has no psychoactive properties and is a safe and effective natural cannabinoid [26]. Moreover, CBD has been approved by FDA for the treatment of children's epilepsy [27] and has shown therapeutic potential in a variety of diseases, particularly inflammation-related diseases [28] and neuroprotective ability [29]. Recently, increasing research has found antioxidant effects of CBD, especially protection of keratinocytes against exposure to UV and H_2_O_2_ [15, 30, 31]. To date, Cuba's review evaluated the feasibility of CBD in the treatment of OM [32]. However, comprehensive experimental data and mechanistic evidence regarding the effectiveness of CBD for OM are lacking.

The only study describing CBD in OM mice reported only a possible tendency with limited significant data [17]. In their study, the weight loss in the CBD group seemed to be lower than that in the 5-FU alone group, but no significant difference was observed. Consistent with their outcome, we first found that CBD significantly reversed the weight loss from Day 4, and the total weight loss was also statistically improved. A previous study found that CBD increased body weight in rats and was associated with reversing intestinal inflammation [33]. In addition, our data demonstrate that CBD improved survival and fecal scores in mice exposed to 5-FU toxicity, while the diarrhea was also an important clinical indicator of intestinal mucosal inflammation, which is consistent with previous results.

Oxidative stress and proinflammatory cytokines are directly involved in mucosal injury secondary to tumor treatment and are key mediators of OM. Therefore, the control of oxidative stress and inflammatory response is essential to avoid harmful pathological reactions [34]. First, ROS production is the initiating factor in the pathological process of OM caused by radiotherapy and chemotherapy, leading to DNA damage and cell death. In this study, we found that CBD significantly suppressed ROS generation in 5-FU-treated mouse tongue tissues and HOK, which is consistent with other reports that CBD reduced the ROS levels of keratinocytes [35], microglia [36], and endothelial cells [16].

ROS-mediated DNA damage directly results in cell apoptosis, which interferes with cell proliferation and the epithelial healing process. ROS reduces the cell viability of keratinocytes and inhibits wound healing in OM [37]. CBD have an antiapoptotic effect on keratinocytes irradiated by ultraviolet light [38, 39]. Considering the antiapoptotic properties of CBD, we evaluated the apoptosis of epithelial cells in mouse tongue tissue by Ki67 staining and TUNEL assay. Flow cytometry was also used to detect HOK apoptosis changes *in vitro*. mRNA expression of the apoptosis-related genes *Bax* and *Bcl-2* was also detected. The results showed that CBD could effectively reduce the apoptosis rates in tongue epithelial cells and HOK cells. Furthermore, CBD promoted the proliferation of epithelial basal cells and HOK and enhanced the healing rates of HOK in a 5-FU-induced environment. In similar research, CBD increases the proliferation and migration of keratinocytes [40] and endothelial cells [41].

Under normal circumstances, the activation of the Nrf2/Keap1/ARE signaling pathway is the main endogenous defense mechanism to reduce ROS production and defend against oxidative stress [42]. Increasing evidence shows that the Nrf2/ARE signaling pathway is closely associated with mucositis pathology and plays a key role in the oxidative stress response during OM progression [10]. Nrf2 is a key transcription factor in oxidative stress defense that is usually located in the cytoplasm and binds with Keap1 protein [43]. After stimulation, Nrf2 separates from Keap1, translocates into the nucleus, and then combines with the antioxidant response elements (AREs) and upregulates the expression of key genes including HO-1 and NQO1 [44]. Oral mucosal cell knockout of Nrf2 failed to induce the synthesis of antioxidant genes [45]. Braun et al. found that the expression of various key players involved in wound healing was significantly reduced in Nrf2 knockout animals [46].

In addition, proteomic studies and transcriptional profiles have found that Nrf2 may be the downstream target of CBD [21, 22]. In our study, we found that CBD upregulated the gene expression of antioxidant enzymes, including HO-1, NQO1, and SOD1, endogenous antioxidants that can eliminate free radicals, which can be reversed by ML385 treatment and Nrf2-siRNA transfection. Western blotting, qRT-PCR, and IF analysis showed that CBD promoted the increase and nuclear translocation of Nrf2, which could explain the increased expression of HO-1 and NQO1 induced by CBD intervention. In UV-irradiated keratinocytes, CBD significantly enhances the activity of antioxidant enzymes by stimulating the transcriptional activity of Nrf2 [47], consistent with our study.

Keap1 is the main regulator of Nrf2 degradation, and it usually binds to Nrf2 in the cytoplasm, promotes stable ubiquitination degradation of Nrf2, and has been considered an indispensable mediator of the Nrf2/ARE signaling pathway [48]. In this study, we found that the protein level of Keap1 was significantly increased in the 5-FU group but decreased in the CBD group, indicating that CBD promoted Keap1 degradation and improved Nrf2 protein stability. This study was the first to identify the interaction between CBD and Keap1 protein. These results demonstrate that CBD regulates both the Nrf2 and Keap1 proteins and downstream antioxidant enzymes and protects against OM via the Nrf2/Keap1/ARE signaling pathways.

The ROS-mediated inflammatory cascade first activates innate immune responses and then produces inflammatory cytokines, which are also the core of OM pathogenesis [11]. Previous studies have found that CBD prevents microglial inflammation by inhibiting ROS/NF-*κ*B-dependent signaling [36]. Consistent with the former study, we found that CBD suppressed the high levels of the inflammatory cytokines TNF-*α* and IL-6. The NF-*κ*B pathway is also an important signaling pathway in OM pathology [10]. Although we did not further study the effect of CBD on the NF-*κ*B pathway in OM, we demonstrated the potential of CBD in inhibiting inflammatory signaling.

The novelty of our study lies in the discovery of beneficial effects and new possible mechanisms of CBD on chemotherapy-induced OM, which is the first in relevant researches. Based on previous studies which conceived the feasibility of CBD application [17, 32], our study, for the first time, revealed that CBD provided chemopreventive effects via the Nrf2/Keap1/ARE signaling pathway. These promising results in antioxidant and anti-inflammation properties endow CBD therapeutic evidence as protective choice for chemotherapy-caused OM.

On the other hand, as the side reaction of oncotherapy, the pathological mechanism of OM is still unclear and the symptom management is still not satisfactory. In our research, preclinical studies provided new therapeutic perspectives for CBD. However, further translational medicine studies need to be designed to determine more information about drug activity and mechanism of CBD application for human.

This study also has some limitations. First, the detailed regulatory mechanisms between CBD and Nrf2 activation remain unclear. More research is needed to explore the association between CBD and Nrf2 phosphorylation, the main mode of Nrf2 activation. Second, the interaction of CBD with tumor cells in specific diseases and treatment procedures still needs more animal experiments and clinical trials to measure the ultimate outcomes.

## 5. Conclusions

CBD alleviates chemotherapy-induced OM and protects against the toxicity of 5-FU by improving oxidative stress defense, downregulating mucosal inflammation, promoting cell proliferation, and inhibiting 5-FU-induced apoptosis both in mice and in HOK. Moreover, CBD-activated Nrf2/Keap1/ARE signaling pathways might be the underlying mechanism for OM recovery.

## Data Availability

The original contributions presented in the study are included in the article and supplementary material; further inquiries can be directed to the corresponding author.

## Supplementary Materials

Supplementary 1 Figure S1: Cytotoxicity analysis and transcription expression of important genes in 5-FU-, H_2_O_2_-, and LPS-treated HOK cells. (a) The mRNA expression levels of *TNF-α* and *IL-6* after 5-FU (10 *μ*g/mL) and cotreatment with CBD (0.1-10 *μ*M) intervention. ^∗^*P* < 0.05*vs.* the 5-FU alone group. (b) The cell viability (%) of HOK cells treated with H_2_O_2_ for 24 hrs and 48 hrs (50-400 *μ*mol/L). (c) The mRNA expression levels of *SOD1* and *HO-1* after H_2_O_2_ (100-400 *μ*mol/L) intervention. (d) The cell viability (%) of HOK cells after 24 hrs and 48 hrs of LPS (0.5-20 *μ*g/mL) treatment. (e) The mRNA expression levels of *TNF-α* and *IL-6* after 5-FU (1-20 *μ*g/mL) intervention. (f) The mRNA expression levels of *TNF-α* and *IL-6* after LPS (0.5-20 *μ*g/mL) intervention. *β*-Actin was used as the internal control of qRT-PCR. Data are expressed as the means ± SD. ^∗^*P* < 0.05*vs.* the Vehicle group.

Supplementary 2 Figure S2: original blots of western blots for Figure 5(c).

Supplementary 3 Figure S3: original blots of western blots for Figure 6(b).

Supplementary 4 Figure S4: original blots of western blots for Figure 7(a).

Supplementary 5 Figure S5: original blots of western blots for Figure 8(b).

Supplementary 6 Table S1: sequences of primers used for qRT-PCR. Table S2: the grades of fecal score. Table S3: the descriptions of coat score.
