# Supplementary figures and images for: Protective Effects of Cannabidiol on Chemotherapy-Induced Oral Mucositis via the Nrf2/Keap1/ARE Signaling Pathways
Source: Oxid Med Cell Longev. 2022 May 25;2022:4619760. doi: 10.1155/2022/4619760 (PMC9165619; doi:10.1155/2022/4619760)

Figure S1

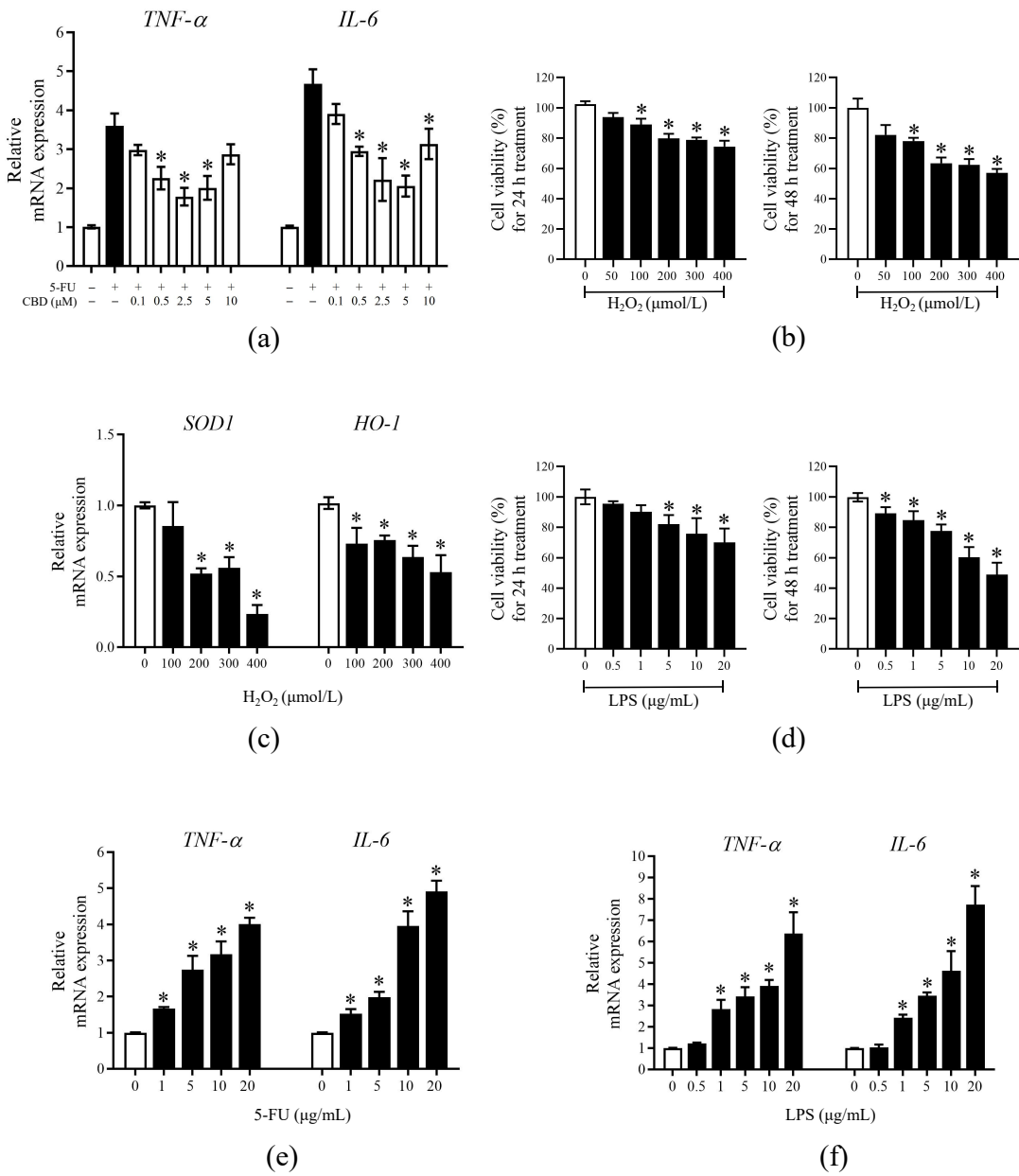

Supplement: Supplementary 1 — Figure S1: Cytotoxicity analysis and transcription expression of important genes in 5-FU-, H2O2-, and LPS-treated HOK cells. (a) The mRNA expression levels of TNF-α and IL-6 after 5-FU (10 μg/mL) and cotreatment with CBD (0.1-10 μM) intervention. ∗P < 0.05vs. the 5-FU alone group. (b) The cell viability (%) of HOK cells treated with H2O2 for 24 hrs and 48 hrs (50-400 μmol/L). (c) The mRNA expression levels of SOD1 and HO-1 after H2O2 (100-400 μmol/L) intervention. (d) The cell viability (%) of HOK cells after 24 hrs and 48 hrs of LPS (0.5-20 μg/mL) treatment. (e) The mRNA expression levels of TNF-α and IL-6 after 5-FU (1-20 μg/mL) intervention. (f) The mRNA expression levels of TNF-α and IL-6 after LPS (0.5-20 μg/mL) intervention. β-Actin was used as the internal control of qRT-PCR. Data are expressed as the means ± SD. ∗P < 0.05vs. the Vehicle group. [file 4619760.f1.pdf]

Figure S2

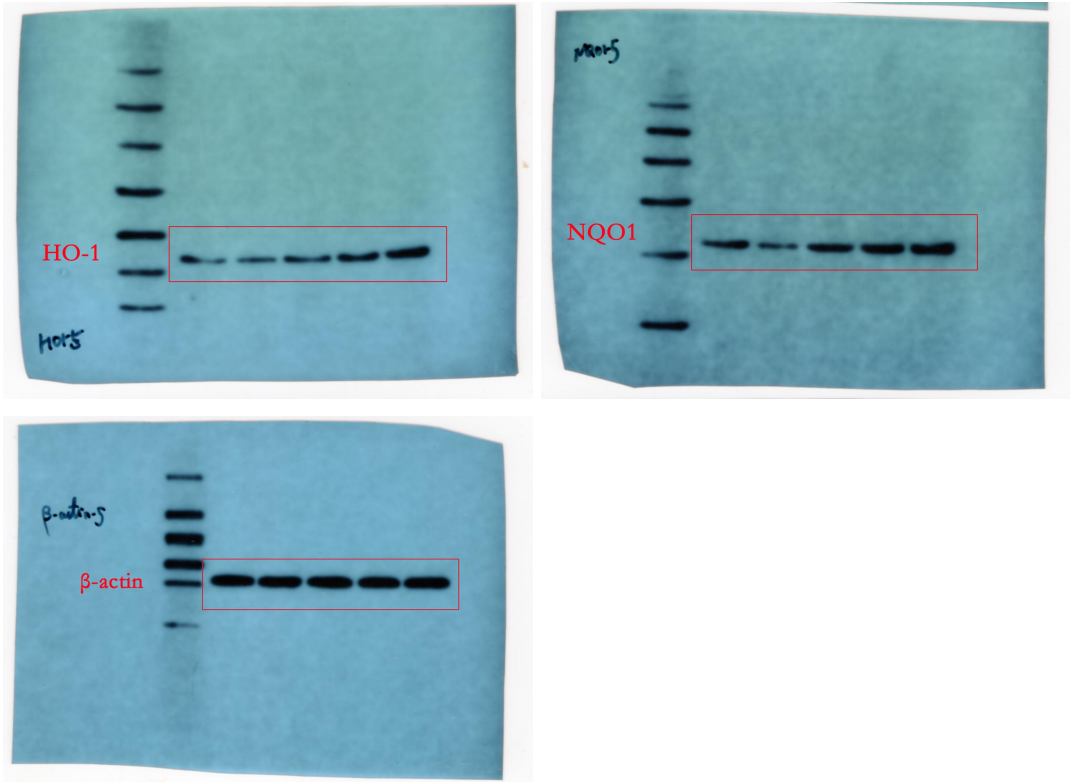

Supplement: Supplementary 2 — Figure S2: original blots of western blots for Figure 5(c). [file 4619760.f2.pdf]

Figure S3

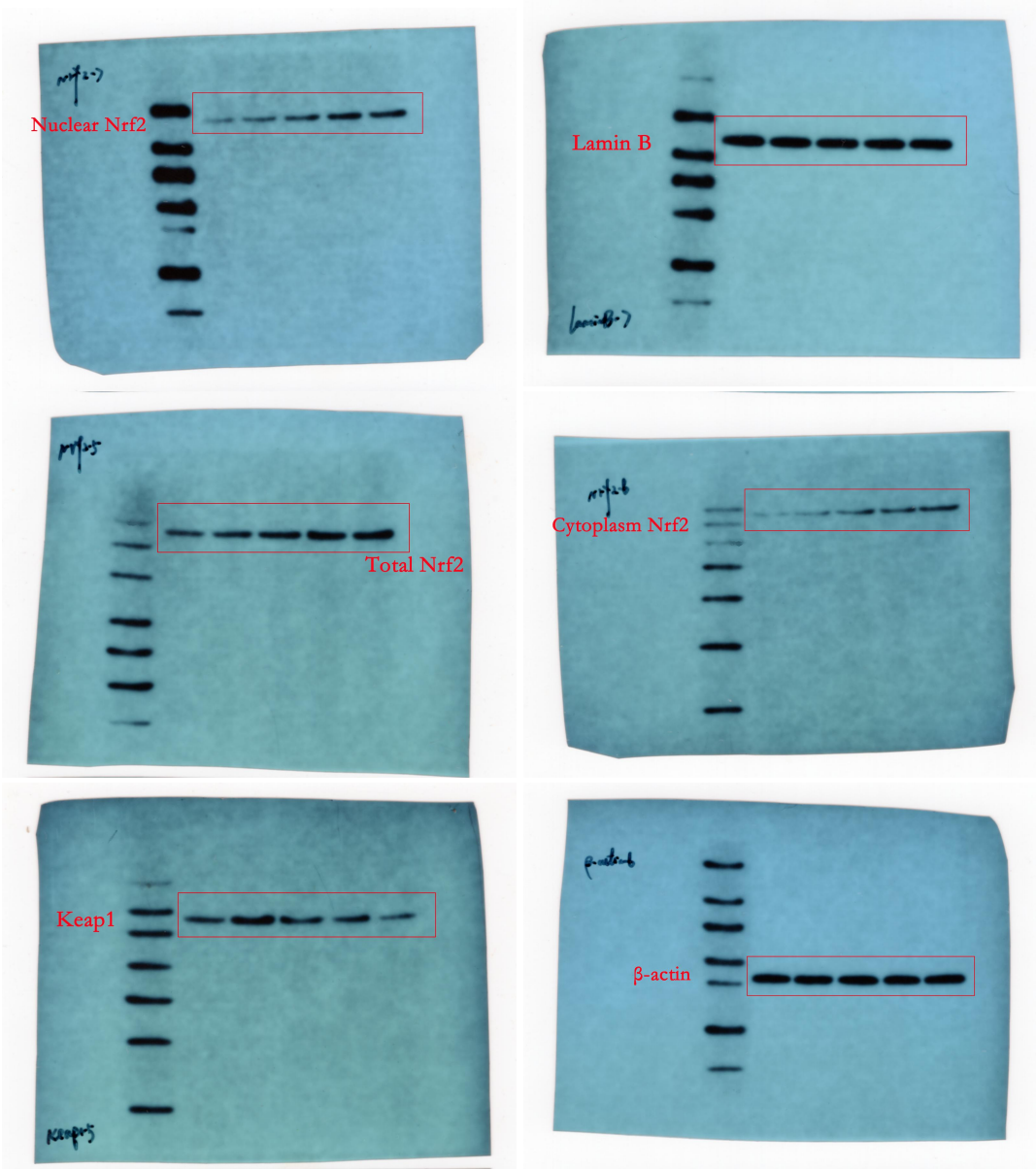

Supplement: Supplementary 3 — Figure S3: original blots of western blots for Figure 6(b). [file 4619760.f3.pdf]

Figure S4

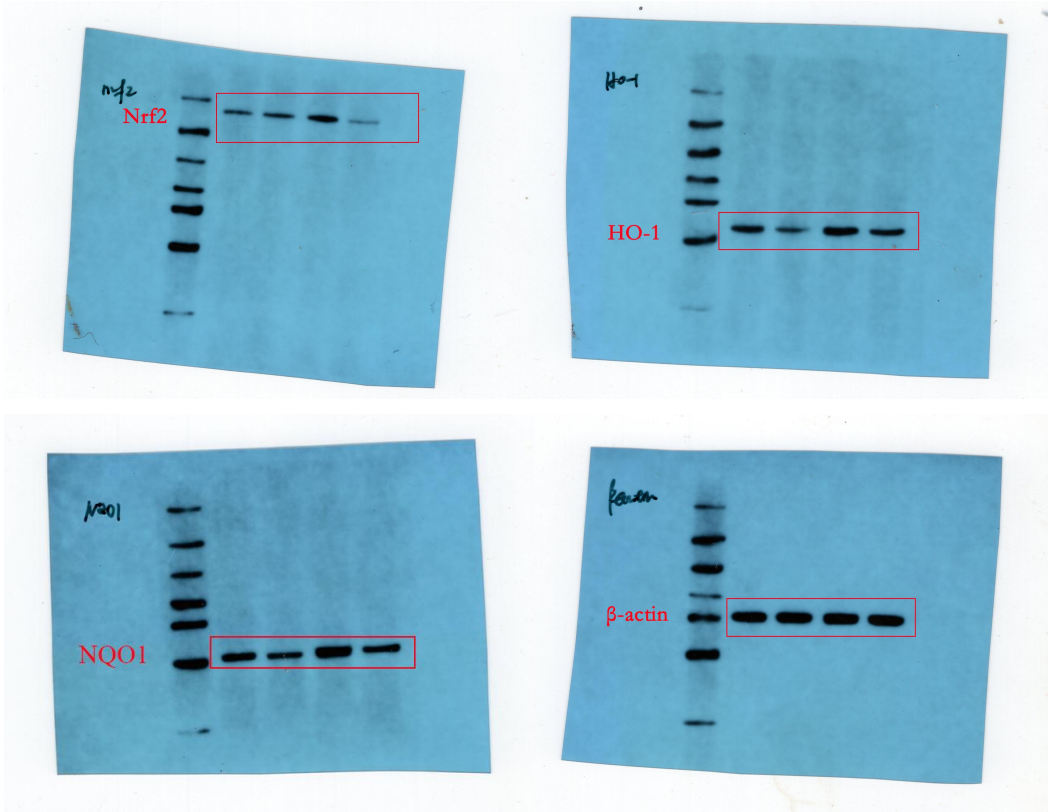

Supplement: Supplementary 4 — Figure S4: original blots of western blots for Figure 7(a). [file 4619760.f4.pdf]

Figure S5

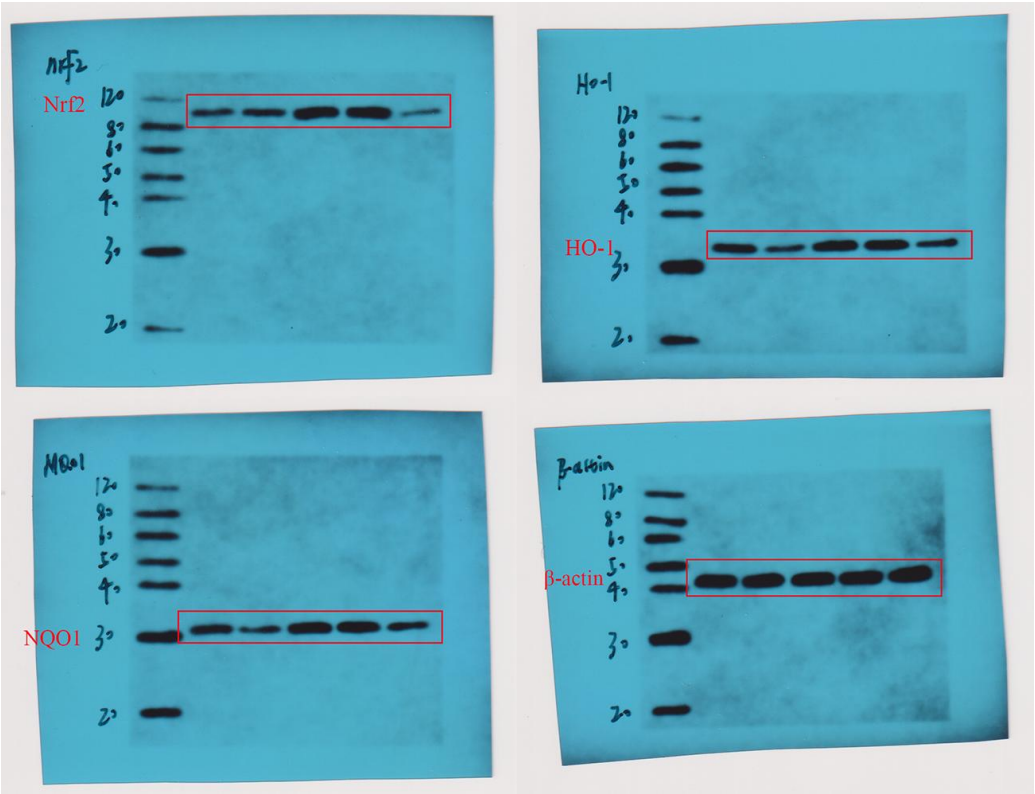

Supplement: Supplementary 5 — Figure S5: original blots of western blots for Figure 8(b). [file 4619760.f5.pdf]
