# Supplementary material for: Protective Effects of Cannabidiol on Chemotherapy-Induced Oral Mucositis via the Nrf2/Keap1/ARE Signaling Pathways
Source: Oxid Med Cell Longev. 2022 May 25;2022:4619760. doi: 10.1155/2022/4619760 (PMC9165619; doi:10.1155/2022/4619760)
Supplement: Supplementary 6 — Table S1: sequences of primers used for qRT-PCR. Table S2: the grades of fecal score. Table S3: the descriptions of coat score. [file 4619760.f6.docx]

| Table S1. Sequences of primers used for qRT-PCR. | | | |
| --- | --- | --- | --- |
|  | Gene | Forward primer sequence (5′–3′) | Reverse primer sequence (5′–3′) |
| Mus musculus | β-actin | CTACCTCATGAAGATCCTGACC | CACAGCTTCTCTTTGATGTCAC |
|  | SOD1 | TGTCCATTGAAGATCGTGTGAT | TCATCTTGTTTCTCATGGACCA |
|  | NQO1 | GAAGACATCATTCAACTACGCC | GAGATGACTCGGAAGGATACTG |
|  | HO-1 | TCCTTGTACCATATCTACACGG | GAGACGCTTTACATAGTGCTGT |
|  | IL-6 | TCTGGAGCCCACCAAGAACGATAG | GTCACCAGCATCAGTCCCAAGAAG |
|  | TNF-α | ATGTCTCAGCCTCTTCTCATTC | GCTTGTCACTCGAATTTTGAGA |
|  | Bax | TTGCCCTCTTCTACTTTGCTAG | CCATGATGGTTCTGATCAGCTC |
|  | Bcl-2 | AGCCCAATGCCCTCCAGAGC | TCTCAAGCCTTCACGCAAGTTCAG |
| Homo sapiens | β-actin | CATGTACGTTGCTATCCAGGC | CTCCTTAATGTCACGCACGAT |
|  | Bax | CGAACTGGACAGTAACATGGAG | CAGTTTGCTGGCAAAGTAGAAA |
|  | Bcl-2 | GACTTCGCCGAGATGTCCAG | GAACTCAAAGAAGGCCACAATC |
|  | HO-1 | CCTCCCTGTACCACATCTATGT | GCTCTTCTGGGAAGTAGACAG |
|  | NQO1 | AAGCCGCAGACCTTGTGATATTCC | CTCTCCTATGAACACTCGCTCAAACC |
|  | SOD1 | ATCCTCTATCCAGAAAACACGG | GCGTTTCCTGTCTTTGTACTTT |
|  | TNF-α | AAGGACACCATGAGCACTGAAAGC | AGGAAGGAGAAGAGGCTGAGGAAC |
|  | IL-6 | CACTGGTCTTTTGGAGTTTGAG | GGACTTTTGTACTCATCTGCAC |
|  | Nrf2 | CCCAGCACATCCAGTCAGAAACC | AGCCGAAGAAACCTCATTGTCATCTAC |
|  | Keap1 | ATTCAGCTGAGTGTTACTACCC | CAGCATAGATACAGTTGTGCAG |

Abbreviations: qRT-PCR, quantitative real-time polymerase chain reaction; SOD1, superoxide dismutase 1; NQO1, NAD(P)H quinine oxidoreductase 1; HO-1, heme oxygenase-1; IL-6, interleukin-6; TNF-α, tumor necrosis factor-α; Bax, BCL2 associated X; Bcl-2, B-cell lymphoma-2; Nrf2, nuclear factor erythroid 2-related factor 2; Keap1, Kelch-like ECH-associated protein 1.

Table S2. The grades of fecal score.

| Score | Severity | Description |
| --- | --- | --- |
| 0 | normal | normal or absent stools |
| 1 | slight | slightly wet, and soft stools |
| 2 | moderate | wet, and unformed stools with perianal staining of the coat |
| 3 | severe | watery stools with severe perianal staining of the coat |

Table S3. The descriptions of coat score.

| Score | Description | Sites |
| --- | --- | --- |
| 0 | clean, neat, and smooth hair | head, neck, back, abdomen, and back claw (*n*=5) |
| 1 | messy, fluffy, and greasy hair |  |
